# An indigenous approach to explore health-related experiences among Māori parents: the Pukapuka Hauora asthma study

**DOI:** 10.1186/1471-2458-13-228

**Published:** 2013-03-15

**Authors:** Bernadette Jones, Tristram R Ingham, Fiona Cram, Sarah Dean, Cheryl Davies

**Affiliations:** 1 University of Otago Wellington, PO Box 7343, Wellington, New Zealand; 2 Katoa Ltd., PO Box 105611, Auckland, 1143, New Zealand; 3 University of Exeter, Devon, UK; 4 Tu Kotahi Māori Asthma Trust, Wellington, New Zealand

**Keywords:** Māori, Indigenous, Qualitative, Methods, Asthma, Children, Experiences

## Abstract

**Background:**

The prevalence of asthma for Indigenous New Zealand Māori is amongst the highest in the world. Recent evidence shows ethnic differences in asthma symptom prevalence in New Zealand have widened, with asthma symptoms and hospitalisation rates consistently higher for Māori across all age-groups, especially children and adolescents. This paper: outlines our qualitative, longitudinal research exploring the practical issues Māori children and their families face trying to achieve optimum asthma outcomes; details the research methods used within this study; and discusses the process evaluation findings of the features that made this approach successful in engaging and retaining participants in the study.

**Methods:**

Thirty-two Māori families were recruited using a *Kaupapa Māori* (Māori way) Research approach. Each participated in a series of four in-depth interviews that were carried out at seasonal intervals over the course of one year. Families also took part in an interviewer-administered questionnaire and participated in a Photovoice exercise. All interviews were digitally recorded, transcribed verbatim and independently coded by two researchers. The research team then conducted the analysis and theme development. The questionnaires were analysed separately, with explanations for findings explored within the qualitative data.

**Results:**

The methodology produced a 100 percent retention rate of the participating families over the course of the follow-up. This was attributed to the research collaboration, the respectful research relationships established with families, and the families’ judgement that the methods used enabled them to tell their stories. The acceptability of the methodology will add to the validity and trustworthiness of the findings.

**Conclusion:**

Given the extent and persistence of ethnic disparities in childhood asthma management, it is imperative that an indigenous approach be taken to understanding the core issues facing Māori families. By conducting community-partnership research underpinned by an indigenous methodology, and employing a range of appropriate methods, we have successfully recruited and retained a cohort of Māori families with experiences of childhood asthma. We aim to make their voices heard in order to develop a series of culturally relevant interventions aimed at remediating these disparities.

## Background

Asthma is the commonest chronic health condition affecting New Zealand (NZ) children, with prevalence rates amongst the highest in the world [1,2]. There has been considerable epidemiological research undertaken to explore risk factors for asthma in NZ, including a number of large international surveys [3-5], and longitudinal birth cohort studies [6-8]. Relatively few studies have investigated issues specific to Māori, the indigenous population of NZ [9].

Māori have long been known to suffer significant disparities of asthma in terms of morbidity, mortality, disease severity, and management [10,11]. Time-trends indicate that the prevalence of asthma has decreased for New Zealand European populations over the past few decades [4], but this reduction has not occurred for Māori [12]. Māori children suffer higher severity of asthma symptoms when presenting to health care providers for routine or acute care [13], and have significantly higher rates of hospitalisation [14]. They are less likely to have been given a peak flow meter or asthma action plan, and fewer are prescribed regular inhaled corticosteroids [14,15].

In 1991, a ministerial review ‘*He Mate Huango*: Māori Asthma Review’ was conducted to consider and address the burden of asthma for Māori [16]. Recommendations of the report included: the need for more effective involvement of Māori in the planning and delivery of asthma care; a need for improved access to health care; that Māori be involved in all aspects of the education process relating to asthma and asthma management; that appropriate information and education materials about asthma be made available; and that cultural safety education be included in the training of health workers. A review published a decade later updating the original report indicated that most of the initially described disparities persisted, and that few of the recommendations had been successfully implemented [17]. The review went on to note that “continued research in this area is critical given that so little, still, has been done specifically looking at asthma in Māori.” [Ibid, Pg. 90]

In order to address overall disparities for Māori, it is essential to understand the health experiences of Māori in childhood, because ultimately the health of adolescents and adults are determined in these early years [11,18]. Moreover, engagement with the whole family is important because of the central role that *whānau* (family) have in Māori culture and in health decision-making [19]. Future interventions to address these disparities need to be developed in partnership with, and delivered in ways that meet the needs of, the people concerned [9].

Internationally a wide range of Indigenous approaches to research with Indigenous communities have been described [20-23]. Despite their distinctive identities, these Indigenous methodologies have a common philosophical basis in their efforts to reflect the unique epistemologies (ways of knowing), axiologies (ways of doing), and ontologies (ways of being) of the indigenous population [24]. Indigenous methodologies make visible what is meaningful and logical in respect of indigenous peoples’ own understanding of themselves and the world [25]. Such approaches do not exclude the use of a wide range of methods (including western), but instead demand the interrogation and/or adaptation of these methods with respect to: cultural sensitivity; cross-cultural reliability; outcome utility; and other such measures, to meet the needs of the indigenous participants and community [22,26].

The *Pukapuka Hauora* (Healthy Lungs) Asthma Study aimed to collect and understand the insights of Māori parents, and their children, exploring their day-to-day realities, beliefs about asthma management as well as their experiences and challenges in achieving optimum asthma outcomes. In order to collect this information in a culturally appropriate manner and achieve meaningful results it was necessary to develop a process that would be both acceptable to, and respectful of, this indigenous community.

This paper describes an indigenous-based, qualitative study, including the *Kaupapa Māori* (Māori way) research paradigm that framed this study, and the specific methods employed. This description is accompanied by process evaluation findings, including our critical reflection on the aspects that made this approach successful in engaging and retaining participants in the study.

## Methods

### Methodology

This was a longitudinal, qualitative, exploratory field study utilising a *Kaupapa Māori* Research paradigm and Interpretive Phenomenological Analysis (IPA) methodology.

#### Kaupapa Māori Research

A *Kaupapa Māori* Research (KMR) paradigm is an emancipatory, Indigenous research paradigm connected to Māori philosophy and principles [20,27,28]. KMR takes for granted the validity and legitimacy of Māori and the importance of Māori language and culture with a focus on autonomy over Māori health and well-being [29]. KMR differs from the four dominant paradigms described by Guba and Lincoln [30] in that ‘relationality’ is at the heart of KMR, as it is in other Indigenous research paradigms [31]. In other words, the core of the Māori world rests on genealogical relationships (*whakapapa*) and Māori research is accountable to these relationships. Knowledge, therefore, is for the good of the people rather than individual gain [32].

What KMR has in common with other, transformative research approaches is that KMR is also about making a positive difference for Māori [33]. In health, for example, Community-Based Participatory Research (CBPR) sets out to reduce health disparities through a commitment to university-community research collaborations [34]. A similar agenda is reflected in the research on asthma described here. The research team for the present project consisted of university and community-based Māori and non-Māori researchers, with a community-based Māori Health Provider, a non-government organisation (NGO). Our community research partner, *Tu Kotahi* Māori Asthma Trust (*Tu Kotahi*), provides culturally appropriate asthma services to their community utilising a holistic Māori model of health [19]. Senior *Tu Kotahi* staff were involved throughout all phases of this research.

KMR employs a large variety of traditional and contemporary, qualitative and quantitative research methods [20]. These are drawn upon appropriately and creatively so that the methods, and often their methodological traditions, are in alignment with KMR [35,36]. Each of the methods used in this study was subjected to scrutiny to ensure their compatibility with KMR, including their ability to facilitate Māori families’ sharing of their experiences in a culturally appropriate and safe way [37].

#### Interpretive Phenomenological Analysis

Interpretive Phenomenological Analysis (IPA) came to prominence in the mid-1990s as a qualitative research approach within health psychology [38]. By taking a hermeneutic approach to studying how people experience and interpret an event or phenomenon, IPA aims to explore and learn about participants’ worlds in depth [39]. IPA therefore allows researchers to develop an analytical interpretation of participants’ accounts that are grounded in (but may go beyond) the participants’ own sense-making [40]. A key component of this is the acknowledgement of the importance of participants’ social and cultural context [40].

While IPA had not previously been used in research with Māori, phenomenology (on which IPA is based) [41] has been used successfully with other indigenous peoples in the USA and Canada [21]. Phenomenology aligns with holistic Indigenous cultural values; as a research method it elicits implicit meanings of indigenous culture and has assisted with recording the essence of experiences of indigenous societies [21].

Our motivation to use IPA as a research method was its potential acceptability to Māori research participants, and its ability to elicit and interpret Māori experiences. Our preliminary interrogation of the method pointed to its capacity to privilege Māori interpretations of *whānau* (family), community and *Iwi* (tribal) values, thereby confirming our impression that IPA would enable us to elicit, interpret and appropriately represent Māori experiences of parenting a child with asthma [37].

An additional component to the research design was the longitudinal nature of the study, involving four quarterly visits over the course of a year. Qualitative Longitudinal Research (QLLR) is predicated on the investigation and interpretation of change over time and process in social contexts [42]. This QLLR ‘panel study’ design was necessitated to capture the seasonal variations experienced in environmental factors, asthma triggers, control and health-related experiences [Ibid.] [43]. However it also served a dual functionality insofar as working with families over this extended time course was compatible with our KMR approach, and enabled the building of relationships and trust.

### Ethical and Māori community approval

This project underwent a two-stage review process including independent peer review followed by expert committee review prior to being awarded funding from The Health Research Council of NZ. Formal ethics approval to conduct this study was obtained from the NZ Central Regional Ethics Committee (CEN/07/07/048).

*Kaumātua* and *kuia* (tribal elders) from *Tu Kotahi* were also consulted for advice from the inception of this study and permission was sought to conduct this research in partnership with them and their community. In keeping with traditional Māori protocols the launch of the project included a *kaumātua* blessing and introduction to the community at a ceremony held at *Kokiri Marae* (community meeting place), Wellington.

To ensure good clinical practice the researcher took contact details of Māori Health Advocates to all the interviews in order to provide external support for any participant who became distressed whilst discussing their experiences and requested further support. Some participants were referred to health professionals when required for on-going clinical expertise. In addition to participants the researcher’s wellbeing was ensured by following good practice in terms of conducting home based interviews: a mobile phone was carried to home visits and colleagues were informed of the start time, the venue and expected time of completion.

### Sampling and recruitment

#### Sample framework

Participants (index children and their families) were recruited from amongst primary school aged children (usually 5 – 12 years old) of Māori ethnicity (by parental report), living in the Wellington region of NZ - a conurbation of the capital city, Wellington, and the cities of Lower Hutt, Upper Hutt, and Porirua.

We aimed to recruit 32 participants to the study. A retention rate of 80% over the twelve month follow-up was estimated based on other local experience with longitudinal, community-based trials, to ensure 24 families completed the study [44].

A purposive sampling framework was employed to capture a diversity of children, in terms of parental socio-economic status (SES) and the presence or absence of a severe exacerbation within the past three years. A questionnaire-based definition of ‘current asthma’ was used in order to facilitate recruitment and avoid any necessity for participants to undergo eligibility testing (e.g. spirometry). ‘Current asthma’ was defined per our previous studies [45,46] as: a prior history of a doctor diagnosis of asthma, plus either inhaler use in the last 12-months or the presence of parent-reported wheeze in the last 12-months, using standardised International Study of Asthma and Allergies in Childhood (ISAAC) questions [47]. Severe exacerbations were defined as those requiring hospital admission or emergency department presentation [48]. Similarly we chose ‘Community Services Card’ (CSC) eligibility as a proxy measure of socio-economic status. This income-tested card provides subsidised health care and medication charges for low to moderate income earners [49]. CSC status has been shown to be associated with increased health need, as well as relative economic disadvantage [50].

#### Recruitment

Previous research experience within our team demonstrated that Māori participants prefer any initial contact to be in person ‘face-to-face’ (*kanohi-ki-te-kanohi*) [51], and that a ‘snowball’ effect would occur once the community learns about the study [52]. We designed the recruitment strategy in partnership with *Tu Kotahi* who have over two decades experience working with this Māori community. This involved the *Tu Kotahi* community-based Māori asthma nurses verbally approaching potential participants from their case list and using a standardised statement to inform potential participants about the study and ask for permission for a researcher to contact them by phone. A Māori researcher (BJ) then met with participants to give them a full verbal explanation of the study, provide them with an information sheet, and obtain written consent.

### Tools

This section describes the ‘tools’ used with participants, with the following section, ‘procedure’, describing how they were used during the interviews.

The tools or methods utilised aimed for compatibility with Māori oral and visual traditions. Some of these methods were trialled during earlier pilot studies and found to be culturally acceptable [51,53]. The methods used were: semi-structured interviews, Photovoice, lung drawings, verbal asthma history, and an asthma risk-factor questionnaire.

#### Semi-structured interviews

The interview schedule was developed from the semi-structured questions previously tested in our pilot study [51]. The questions were clear and constructed in a format familiar to the sample population [54]. These questions were sufficiently broad in nature to capture individual experiences while also allowing comparisons to be made between participants during the analysis phase [55]. The primary participant was one or both parents/guardians/caregivers of the index child; however the option was available for children and extended family members to participate in a family focus group interview. The interviews were designed to be conducted at three monthly intervals during the year in order to capture periods of altering asthma symptoms and related experiences throughout the different seasons.

#### Photovoice

Parents and children were asked to use Photovoice [56] as a method of data collection. This technique can add validity and reliability to data collection while ‘enlarging’ the remembering [57]. The inclusion of Photovoice and lung drawings (see below) had a dual purpose as these methods were designed to be both culturally appropriate and child-friendly [58].

For Photovoice we gave a disposable camera to participants who were asked to take photographs of whatever they felt was relevant to their experience of managing asthma. It was completely up to the participants to decide when and what photographs to take instead of being passive recipients of images taken by others [56]. Using images in this way can connect a person to an experience visually, adding a different dimension and aiding both the participant and the researcher’s interpretation of a phenomenon. Photovoice use has been documented in a number of health research projects including chronic illness [59,60], although less frequently with children [61,62].

From a cultural perspective the use of images has been described as “bridges between worlds that are more culturally distinct” [57] and was found acceptable by ethnic minorities as a means of establishing a deeper understanding of cultural and social experiences [63]. Photovoice has been used successfully with Indigenous participants helping to make visible their experiences and promote their needs in the healthcare setting [64]. In the NZ context it has been used effectively with Māori youth as an indigenous, community change initiative, addressing the complex issue of family violence [65,66].

#### Lung drawings

Children (and their parents) were asked if they would like to draw a picture of their lungs and describe visually what having asthma means to them. An outline of a child was provided with coloured pens and participants were asked to draw whatever visual representation they liked. Drawings can be well suited to research as an adjunct to other social research methods and both the drawing and the participants explanations provide another source of data that enriches the analysis [67]. Drawings have also been commonly used in research with young children [68], as a different way of collecting information, especially for concepts or experiences that may be difficult to clearly articulate [69,70].

#### Asthma history, risk-factor questionnaire and field diary

A researcher administered (BJ) asthma history and risk-factor questionnaire was undertaken with all participants. This was developed based on a number of sources which had been tested for validity and acceptability, including: the ISAAC Phase II questionnaire [47], the New Zealand Census 2006 [71], the ‘*He Kainga Oranga* Healthy Housing Programme’ [72], and the NZ Index of Socioeconomic Deprivation for Individuals (NZiDep) [73]. The questions were selected to provide information on a range of topics including: asthma severity, asthma management, environmental risk factors, housing characteristics (ownership, crowding, heating, dampness etc.), and socio-economic indicators.

Observational data were also recorded by the interviewer (BJ) in the form of field diary notes depicting her observations and interpretations immediately following completion of the interviews, as well as reflections on the research process and any limitations and sentinel events. These reflections were linked with the responses during data analysis.

### Procedure

All interviews were audiotaped and transcribed verbatim. Interpreter services were available but not required as all participants spoke both English and Māori languages. All participants were assigned a pseudonym to ensure anonymity for the purposes of transcribing and dissemination of results. A printed transcript was available for all participants with the researcher giving a verbal summary at each subsequent visit. This summary led to further discussion and clarification until all participants were satisfied with the accuracy of the researcher’s interpretations.

#### Interview 1

In the first interview the researcher (BJ) focused on *Whakawhanaungatanga* or building the relationship with each family. *Whakawhanaungatanga* is a key concept for Māori and is embedded within the Māori way of conducting all aspects of interacting with people [74]. While it is initiated at the start of any proceedings the relationship building continues throughout all encounters establishing a mutual trust and sharing of information [37]. Once the researcher had developed a rapport she began the inquiry into understanding parents’ perspectives of caring for a child with asthma including both the challenges and the aspects of management that were useful.

A disposable camera was given to the parent(s) at the end of this first interview with the researcher providing detailed information including: camera use, consent, privacy issues and ownership of any photographs. Informed consent and ethical issues around the use of photographs are well documented by users of Photovoice particularly with vulnerable populations [75]. Permission was sought from participants to use the images for academic publications, conferences and for teaching purposes with medical and nursing students. Parents and children selected photographs to be used for the research with one parent declining the use of Photovoice due to concerns regarding potential misuse of photographs on the internet.

Parents were encouraged to photograph anything that they felt was important about their child’s asthma management experience. While researchers avoided giving explicit instructions as to what photographs to take, a general explanation was given as to potential ways to use the camera to capture important experiences. For example, photographs of environmental factors that exacerbate the child’s asthma (pollen producing plants, mould on windows); management strategies that are used (dust reduction measures such as regular vacuuming, changing bed linen); use of medication (inhalers, spacers); visiting clinics; the people involved in helping the child to manage asthma etc. The researcher arranged to collect the camera and have the film developed in time for the photographs to be used for facilitating discussion at follow-up interviews.

#### Interview 2

The second interview began with *Whakawhanaungatanga* and then focused on themes emerging from the initial interview and events occurring during the past season. This was seen as an opportunity to revisit issues that had been discussed by parents at the first visit and to talk in-depth about aspects of asthma relevant to them. This process was repeated at each subsequent interview. The cameras from participants were collected at this visit, the film developed into both hard and digital copies. Parents were also offered the use of a second camera if they chose. Specific issues illustrated by other participants were discussed in general terms and parents asked to comment on whether these experiences were relevant to them. This was useful as a prompt to generate further in-depth discussion about whether they had similar or different experiences with these emerging issues.

#### Interview 3

A hard copy of the photographs was compiled into an album and given to participants at the third visit. Parents and children present were asked to comment on each photograph and its relationship to their asthma management. Questions at this interview focused on how the child’s asthma had been over the preceding season and highlighted some key topics discussed at the previous interview. Participants were asked what advice they might give to other parents who were newly faced with caring for a child with asthma. This was asked in an effort to understand their individual priorities or concerns when learning to manage asthma. Additionally at this interview both parents and children were given an outline figure of a child and asked to draw a representation of how they visualized their lungs.

#### Interview 4

The fourth and final interview discussed the asthma management over the preceding season including key issues talked about by the participant in the previous interview. This was another opportunity to feedback researcher analysis and interpretations and to ensure participants had the opportunity to edit or alter these interpretations ensuring the data were an accurate representation of what the participant had discussed throughout the study.

The asthma history and risk-factor data were collected by the researcher as part of a questionnaire which also served as a useful interview prompt that enabled participants to ask questions about asthma triggers, risk factors, and medications as well as for the purposes of characterising the participants in terms of asthma history. The semi-structured interview questions also included a focus on what immediate changes they would like to see in asthma management for their child. Finally feedback was sought regarding participant’s experience in the study, what (if anything) they liked most and least about the study, and whether they would prefer changes to the design and/or implementation of the research process.

### Data analysis

The method being used for analysis was Interpretive Phenomenological Analysis (IPA), which has been described as an iterative and inductive cycle [40]. This involves line-by-line analysis of each transcript, followed by the identification of emergent themes. A ‘dialogue’ between the researchers, the transcripts, and their theoretical knowledge of what these concerns might mean for participants leads in turn to the development of a more interpretive account. IPA acknowledges the researcher’s involvement in this analytical process, through which the researcher tries to make sense of the participant who in turn is trying to explain their own experiences [40]. A summary of emergent themes was produced with transcript extracts or photographs that illustrated each theme. Incorporated within the layers of analysis are a number of other robust verification procedures that aid in establishing the trustworthiness of the analysis.

### Validity

Multiple strategies were used to ensure data reliability and validity [76]. Triangulation, a procedure by which researchers search for convergence among multiple and different sources of data in order to form themes or categories, was a central tool in this process [77].

Descriptive validity included the use of ‘investigator’, ‘method’, and ‘data’ triangulation processes [76]. ‘Method triangulation’ was undertaken using the transcripts, Photovoice, and lung drawings as well as field notes to provide a rich collection of data [65]. ‘Data triangulation’ occurred through the inclusion of other family members in many of the interviews, and at different times, and this offered additional perspectives on the issues discussed [78]. ‘Investigator triangulation’ included discussion within the team about the themes and interpretation of explicit and implicit meanings. Collective agreement was then reached about the extent to which data represented themes and interpretations, and how these fitted within the broader cultural and political contexts [79].

Interpretive validity was ensured through participants being given a summary of the previous interview and having the opportunity to comment, give feedback, redirect any interpretations, or edit their transcripts. The draft findings were presented to participant families and community partner at the completion of the study (BJ,TI,CD) [80]. Families were invited to have input into editing and shaping these results and ensuring they represented valid, accurate, meaningful interpretations of their experiences.

‘Extended fieldwork’ and ‘peer review’ were used as key strategies to contribute to the theoretical validity of the findings, so they would be both “credible and defensible” [76]. Fieldwork began with an extensive consultative engagement process [37], and a deeper understanding of the issues, concerns, and context of the community was developed through the conduct of a theme development pilot [51]. Greater insights were crystallised with prolonged involvement in the community over the course of the study, largely facilitated by the longitudinal nature of the participant interviews. Further contributing to the theoretical validity was our peer review process by which researchers independent from data gathering, who were experienced in IPA and/or asthma management, reviewed, discussed, and challenged the themes generated.

## Results

### Participant characteristics

The socio-demographic profiles and health characteristics of participants are shown in Table 1. Thirty-two families were recruited to the study. Index children ranged from ages 4 to 11 years (median=7 years), with 20 (63%) boys. In the previous twelve months, over half of the children (56%) had experienced more than 12 asthma attacks, twenty children (63%) had speech-limiting wheeze, and twenty-one children (66%) had been hospitalised for asthma. Six children (19%) had not been given a peak flow meter. Additionally six parents (19%) reported that they had never been given a written asthma action plan for their child, three of whom had previously been admitted to hospital (data not shown in table).

**Table 1:** Participant demographic characteristics

**Characteristics (N=32)**	**n**	***%***
**Index child demographics**		
Gender (male)	20	*(63%)*
Age in years (median, (range))	7	*(4–11 yrs.)*
**Asthma control**		
Ever hospitalised for asthma? (Yes)	21	*(66%)*
Speech limiting wheeze in the last 12 months? (Yes)	20	*(63%)*
Attacks of wheezing in the last 12 months:		
1-3 attacks	5	*(16%)*
4-12 attacks	9	*(28%)*
> 12 attacks	18	*(56%)*
**Main caregiver**		
Age in years (median, (range))	35	*(23–59 yrs.)*
Secondary School Education:		
No NZ qualification (<3 years)	13	*(41%)*
Minimum NZ qualification (3 years)	7	*(22%)*
Higher NZ qualification (4–5 years)	11	*(34%)*
Overseas secondary school qualification	1	*(3%)*
Employment Status:		
Paid employment	19	*(59%)*
Stay-at-home parent/ caregiver	12	*(38%)*
Student	1	*(3%)*
**Socioeconomic position**		
Community Service Card Eligibility	26	*(81%)*
NZiDep Index [73]:		
0 deprivation characteristics	1	*(3%)*
1 deprivation characteristic	4	*(13%)*
2 deprivation characteristics	5	*(16%)*
3 or 4 deprivation characteristics	3	*(9%)*
5+ deprivation characteristics	19	*(59%)*
Home Ownership (with or without mortgage)	15	*(47%)*
Household Crowding:		
1-2 people per bedroom	25	*(78%)*
3 or more people per bedroom	6	*(19%)*
No-response	1	*(3%)*

The age of the main caregiver ranged from 23–59 years (median=35 years). Over half (59%) were in paid employment, although a majority (81%) were eligible for a community services card, indicating relative economic disadvantage. Over half of the families (59%) experienced five or more of the deprivation characteristics comprising the NZiDep Index [73].

Nearly half of the families (47%) owned their own home (with or without a mortgage), with 25 (78%) of the families having 1–2 people per bedroom.

Twenty-one (66%) main caregivers involved extended family members in one or more of the interviews. Additional family members who contributed to these interviews included: siblings, aunts, and grandparents of the index child, along with partners and friends of the main caregiver. All 32 participating families remained for the duration of the study, with 127 of the 128 interviews completed within the scheduled visit windows.

### Participant feedback

Feedback from the families who participated (given both directly to researchers and independently through staff at *Tu Kotahi*) indicated they had enjoyed their involvement in the research project, finding the methods to be acceptable and appropriate:

“I liked the sharing, the interviews and the visits, it’s been good.”

“You’ve actually been quite supportive of how our lifestyle’s been and you understand if we can’t make an appointment… not giving up on [us] …you’ve worked with our lifestyle which is great, that makes my job even easier.”

“The camera was a great idea, so no I can’t think of anything that I would want to change”

Part of participants’ enjoyment stemmed from their heightened awareness of, and knowledge about, their child’s asthma. This resulted from the opportunity to reflect on their experiences with the researcher during their interviews, and also the researcher’s ability to respond to questions they had about asthma:

“I like the questions that you’ve asked and being able to discuss things that have made me think about different ways I could have done things… especially with the smoking thing for me… I notice things more now about what I do and how I react.”

“I really enjoyed it. It’s given me a bit more insight into what I am and what I’m doing and … what I’m aware of, maybe more conscious when I think of asthma in our house.”

The research also highlighted for participants that they were not alone in their experiences, and this brought comfort and a feeling of support to many:

“My experience personally has been an eye opener because I’ve heard you mention, we’re not alone in this…as a mother you live it and you don’t stop to think that there’s other people out there in the same predicament…”

“it’s a good support for all families with asthmatic children that are dealing with this sickness to be honest…and I’ve actually enjoyed your company”

Of particular note was that many participants felt that they were ‘understood’ by the researcher (BJ). Her ability to identify with their perspectives as a Māori, a mother, and someone with personal experience managing asthma, was felt by many to make it easier for them to relate and share their experiences:

“it’s been good for me to be able to talk with somebody about it as well. Not all people get it. So you and I get it because we’ve had asthma. We’ve had our own experience of it… It’ll be harder if you’re speaking to somebody who’s never had it ‘cos how are they really going to know?”

Several participants signalled their willingness to be involved in further research studies undertaken by the research team:

“I’m actually quite happy with it you know and I’d sign up for another one if another one came along.”

## Discussion

As part of an ethical and respectful approach, Indigenous methodologies need to explicitly include cultural protocols, values and behaviours as integral components of the research [20]. These factors should be thought about reflexively, declared openly as part of the research design, discussed as part of the final results and disseminated back to the people in culturally appropriate ways in a language that can be understood [Ibid, pg 15]. In accordance with these principles, this paper has documented the procedure of implementing a longitudinal, qualitative study of Māori parents’ experiences of living with a child with asthma, and critically evaluated these processes through feedback from the participants. The key factors in the success of this study, notably the retention of the participants and their good feedback, were (1) the relationship between the participants and the interviewing researcher (BJ), and (2) the support and buy-in from *Tu Kotahi* and the local Māori community.

A well established and mutually beneficial partnership with a Māori health provider *Tu Kotahi* was pivotal to the overall success of this research [37]. The consultation and development of the relationship took a real commitment from both the community and researchers’ perspective. While this involved a considerable investment in staff resources and was time-consuming for both parties it was vital for all concerned to ‘get this right’ prior to the commencement of the study. The research methods and recruitment strategies involved a *kanohi-ki-te-kanohi* (face-to-face) approach and an opportunity for *whānau* (families) to attend a *hui* (meeting) outlining the results of the study.

This establishment of a meaningful community relationship is known as the KMR principle of *kanohi kitea* (the seen face) which advocates that researchers know, and be known by, the community with which they intend to conduct research [81]. There are a number of parallels between this culturally-based paradigm and what Johnson refers to as the validation strategy of ‘extended fieldwork’ [76]. By this he notes that as a researcher “you should spend sufficient amount of time studying your research participants and their setting so that you can have confidence that the patterns of relationships you believe are operating are stable and so that you can understand why these relationships occur”. Our longstanding *a priori* consultative relationship with *Tu Kotahi* and local Māori communities, along with the longitudinal aspects of the follow-up all contributed to the basis for the theoretical validity of this research. This was confirmed by the reciprocal view, of a Māori Provider (CD): “Developing a long-term meaningful relationship with a research institute eventuated in an opportunity for us *[Tu Kotahi]* to have a voice in a project that directly affected our community. We were treated as a significant partner having been involved in all aspects of the project.”

A key indicator of the success of this study was the retention of participants. Such retention depends on a combination of respectful relationships between the researchers and families, and a method that families feel allows them to tell their story. The inclusion of repeat visits with parents and children fitted with cultural expectations of being able to establish a trusted relationship before divulging personal experiences and beliefs. These repeat visits also permitted more accurate collection of information due to seasonal variations and subtle changes that impacted on the management of their child’s asthma. The research experience for families was by no means uniform. Some families opened up at the first interview, others were more reticent and only spoke openly at the third or fourth interview. Even so many of the participating families were amenable to being involved in further research. This was pleasantly rewarding for the research team given the popular myth of Māori being reluctant to be involved in research. This myth likely has its roots in historical experiences of research that could not ‘hear’ Māori voices, let alone promote any evidence-based change based on Māori experience [20,82].

The use of multiple methods, interrogated under KMR, not only added to the robustness and trustworthiness of the data but more importantly these methods were found to be culturally acceptable by the Māori participants. They provided tools that could be used to explore gaps in services, knowledge and understanding from an appreciative inquiry perspective [83] that helps build the research capacity of *whānau* instead from a deficit model. The use of IPA within KMR was found to be highly appropriate for this Māori community as it upheld the rights of the participants throughout the study while allowing for an analysis of societal structures that impacted on their experiences [28].

The semi-structured interview technique was well suited to this study as it allowed the researcher the flexibility to explore the individual experiences of participants who, although all Indigenous, were by no means a homogenous group. A further benefit to the interview process was its capacity to make space for extended family to participate, contribute, and engage. These inputs were seen to enrich the interview content by incorporating different perspectives, as many of these informants were also carers who contributed to the child’s asthma management. Additionally, the involvement of these family members also provided support, encouragement and prompted further discussion. This practice aligned well with Māori/Indigenous frameworks of sharing stories and involving extended family collectives [20,84].

The questionnaire, while predominantly designed to collect an asthma history and demographic data was well received by participants. It served as a useful medium to generate discussion, and acted as a prompt that enabled participants to ask questions about asthma triggers, medication and environmental factors. This reversal of participants questioning the researcher was an example of the power sharing that the use of the KMR paradigm facilitated. Of note from the asthma questionnaire was the level of morbidity experienced by participating *whānau*. Although we purposefully aimed to sample a range of asthma severities, the majority of children studied had poorly controlled asthma with frequent and/or severe symptoms, and hospitalisation. Despite this level of morbidity a number of children did not have an asthma plan or peak flow meter.

While many of the children were too shy to verbally engage at length during the interviews they all welcomed the opportunity to draw and they found the lung representations a fun and engaging way of participating in the research, and generated much discussion about their lungs, and experiences of asthma. Many parents also saw the process of observing the researcher engage with the child whilst completing and discussing the lung drawings, as a non-threatening way of asking their own questions about lungs and asthma that they had previously been uncertain how, and/or too embarrassed, to ask. The discussion topics generated by participants included: basic lung anatomy and physiology; the causes and triggers of asthma; and the subsequent effects of asthma on their body. Overall, this method proved to be a powerful tool which provided a non-threatening context to explore understandings about asthma, and allow participants opportunities to address areas of uncertainty.

Photovoice as a method was embraced by most participants however others were unfamiliar with this approach and struggled to photograph images that were asthma-related. Many requested more detailed explanations and visual examples illustrating a range of items to use before they were confident in taking their own photographs. One of the challenges faced by the researchers was in getting the right balance between providing too little information or being prescriptive and influencing participants’ photograph selections.

An additional challenge in the coding process, for the researchers not directly involved in the interview, was interpreting participants ‘sense-making’ as some of the narratives were not always clearly articulated in the text, but were implicit in the shared understanding between the interviewer and the participant.

Having research credibility in the eyes of Māori participants is not only about good research processes and the right selection of research methods, but also about ensuring that the *taonga* (gift/treasure) of the participants’ stories is shared and well-represented to the wider community. The research team also have a responsibility to advocate for evidence-based change within the health system so that Māori asthma disparities might be reduced.

## Conclusions

We argue that our development of a culturally responsive, KMR longitudinal, qualitative study has allowed a greater and more culturally meaningful engagement with Māori parents, their children and the wider community about experiences of asthma management. As with other Indigenous research, the process itself is considered a vital component of the study and of equal importance as the outcome. The methodology and methods of this study set a benchmark for conducting collaborative, Māori health research that can be used to inform intervention strategies that facilitate Māori health and wellness.

## Abbreviations

CBPR: Community-Based Partnership Research; CSC: Community Services Card; IPA: Interpretive Phenomenological Analysis; ISAAC: International Study of Asthma and Allergies in Childhood; KMR: *Kaupapa Māori* Research; NGO: Non-Governmental Organisation; NZ: New Zealand; NZiDep: New Zealand Index of Socioeconomic Deprivation for Individuals; QLLR: Qualitative Longitudinal Research; SES: Socioeconomic Status; Tu Kotahi: *Tu Kotahi* Māori Asthma Trust

## Competing interests

The authors declare they have no competing interests.

## Authors’ contributions

BJ, TI, SD, and CD made substantial contribution to conception and study design. BJ was the principal investigator and had overall responsibility for the conduct of the study. BJ and TI were involved in data collection. BJ, TI, SD, FC, and CD were involved in coding, analysis, and drafting the manuscript. BJ, TI and FC critically revised the manuscript. All authors read and approved the final manuscript.

## Pre-publication history

The pre-publication history for this paper can be accessed here:

http://www.biomedcentral.com/1471-2458/13/228/prepub
